# The burden of medication overuse headache and patterns of switching and discontinuation among triptan users: a systematic literature review

**DOI:** 10.1186/s12883-021-02451-x

**Published:** 2021-11-02

**Authors:** Alison M. Deighton, Linda A. Harris, Karissa Johnston, Shomari Hogan, Lynn A. Quaranta, Gilbert L’Italien, Vlad Coric

**Affiliations:** 1 Broadstreet HEOR, 201-343 Railway St, Vancouver, BC V6A 1A4 Canada; 2 Biohaven Pharmaceuticals, 215 Church Street, New Haven, CT USA

**Keywords:** Migraine, Triptans, Switching, Discontinuation, Medication overuse headache, Burden

## Abstract

**Background:**

A synthesis of real-world discontinuation and switching patterns among triptan users and rates of acute medication use among patients with medication overuse headache (MOH) is needed to better understand the burden among patients with migraine. The study objectives were to: (1) synthesize rates of switching and discontinuation from triptans; (2) characterize acute medication use among patients with MOH; and (3) describe the associated burden.

**Methods:**

A systematic literature review was conducted, under the Preferred Reporting Items for Systematic Review guidelines, using MEDLINE/EMBASE from database inception to July 2019. The search strategy targeted studies of adults with migraine, and included terms related to migraine and its treatment. Continuous variables were summarized using means, standard deviations, and ranges. Dichotomous and categorical variables were summarized using the number and proportion of individuals.

**Results:**

Twenty studies were included; seven describing patterns of switching and discontinuation among triptan users, and 13 characterizing triptan overuse among patients with MOH. High rates of switching to non-specific acute medications and low two-year retention rates were reported; among US samples switching to opioids at the first refill (18.2%) or after 1-year (15.5%) was frequent. Compared to persistent use of triptans, switchers experienced greater headache related impact and either no improvement or increased headache-related disability. Rates of medication overuse by agent among patients with MOH varied greatly across the included studies, and only one study described factors associated with the risk of MOH (e.g. duration of medication overuse). Medication agent, increased headache frequency (*p* = .008), and increased disability (*p* = .045) were associated with unsuccessful withdrawal; patients overusing triptans were more successful at withdrawal than those overusing opioids or combination analgesics (*P* < .0001).

**Conclusions:**

The evidence summarized here highlights that rates of WCS are low and many patients turn to other acute medication at their first refill. Patients may experience no improvement in disability when switching from one triptan agent to another, or experience increasing disability and/or increasing migraine frequency when turning to traditional acute treatment for migraine. Variability in health care settings, patient severity, and study design contributed to heterogeneity across the synthesis.

**Supplementary Information:**

The online version contains supplementary material available at 10.1186/s12883-021-02451-x.

## Introduction

Migraine is the most debilitating of all health conditions among those younger than 50 years [1]. As the prevalence of migraine is greatest among patients aged 35-49 years of age (i.e. working age) [2, 3], the reduced ability to function during untreated migraine attacks has significant financial impacts at the societal level [4], in the form of absenteeism and presenteeism [5, 6]. Available options for the acute treatment of mild-to-moderate headache include simple analgesics (e.g., aspirin, acetaminophen), nonsteroidal anti-inflammatory drugs (NSAIDS; e.g., ibuprofen, naproxen), opioid analgesics, butalbital-containing analgesic products, and over-the-counter analgesics in combination with caffeine [7, 8]. Following diagnosis, migraine-specific agents may be prescribed for acute treatment, including Serotonin 5-HT1B/1D receptor agonists (triptans,) and Ergot derivatives [7, 9, 10]. Triptans have been considered standard of care [SoC] for the acute treatment of moderate-to-severe migraine for more than 20 years [11].

Approximately 53% of triptan users report at least one unmet need [12], including efficacy and tolerability challenges or adverse events, and 18% of all patients with migraine are contraindicated to triptans due to cardiovascular conditions [11]. Compared to patients who respond to treatment with triptans, patients who do not respond have significantly increased headache frequency, greater impact on health-related quality of life (HRQoL), increased migraine-related disability, and higher direct and indirect medical costs [13, 14]. When an initially prescribed triptan becomes ineffective, it is recommended that a patient try another agent or switch to a combination triptan/analgesic, such as sumatriptan/naproxen, for future attacks [9]. This process of trying another triptan is called within-class switching (WCS), while the process of adding on or switching to a new class of migraine medication is called between-class switching (BCS). A more problematic form of BCS may occur when patients switch to opioids or barbiturates. Although their use may be needed to abort severe attacks, they should generally be avoided as they may reduce patients tolerability to SoC therapies and increase the risk of migraine chronification [9, 10].

Patients with migraine who overuse acute medication are at increased risk for developing chronic migraine as well as medication overuse headache (MOH), a highly disabling secondary headache condition with complex treatment [15–17]. To reduce the risk of MOH, guidelines state that medication use should be limited to 10 days per month for triptans, ergot alkaloids, combination analgesics, or opioids, and 15 days per month for simple analgesics or combination acute medications [15, 18]. Given that many patients experience more frequent migraine, than 10 days per month, there is a need for migraine-specific medication that does not carry a risk of MOH. Currently, the pathophysiology of MOH is not entirely known; however, recent studies have found an increased risk of MOH associated with certain classes of acute medication such as triptans [19, 20]. The treatment of MOH is complex; educating patients about their MOH followed by treatment with preventive medication and withdrawal (i.e. discontinuation of the overused medication) is recommended [21, 22].

Three oral medications for the acute treatment of migraine have recently been approved by the United States Food and Drug Administration: lasmiditan, a 5-hydroxytryptamine -1F (5-HT1F) receptor agonist, and two CGRP receptor antagonists, rimegepant ODT and ubrogepant [9]. The American Headache Society (AHS) recommends that these novel acute medications be considered in patients who have contraindications to the use of triptans or who have failed to respond to, or tolerate, at least two oral triptans [8, 23]. All of the novel medications will fill the need for patients who are contraindicated to the use of triptans; however, only the two CGRP receptor antagonists will not carry a risk of MOH [6, 24, 25] as the prescribing information for lasmiditan contains a warning regarding MOH [24, 26–28].

While reasons for intolerability and persistence rates among triptan users have been described [29, 30], to the best of our knowledge a synthesis on the evidence of the burden of MOH is lacking [17, 31, 32]. A synthesis of real-world discontinuation and switching patterns among triptan users is needed to better understand the burden experienced by triptan non-responders with increased risk of developing MOH, who must experience treatment failure across multiple cycles of migraine medications before being eligible for novel therapies under the current AHS guidelines. The objectives of this systematic review (SLR) were to: (1) synthesize real-world estimates of the number of triptan users, globally, who switch between various triptans or discontinue triptans for other therapies; (2) characterize acute medication use among patients with MOH; and (3) describe the associated burden.

## Methods

**Fig. 1:**
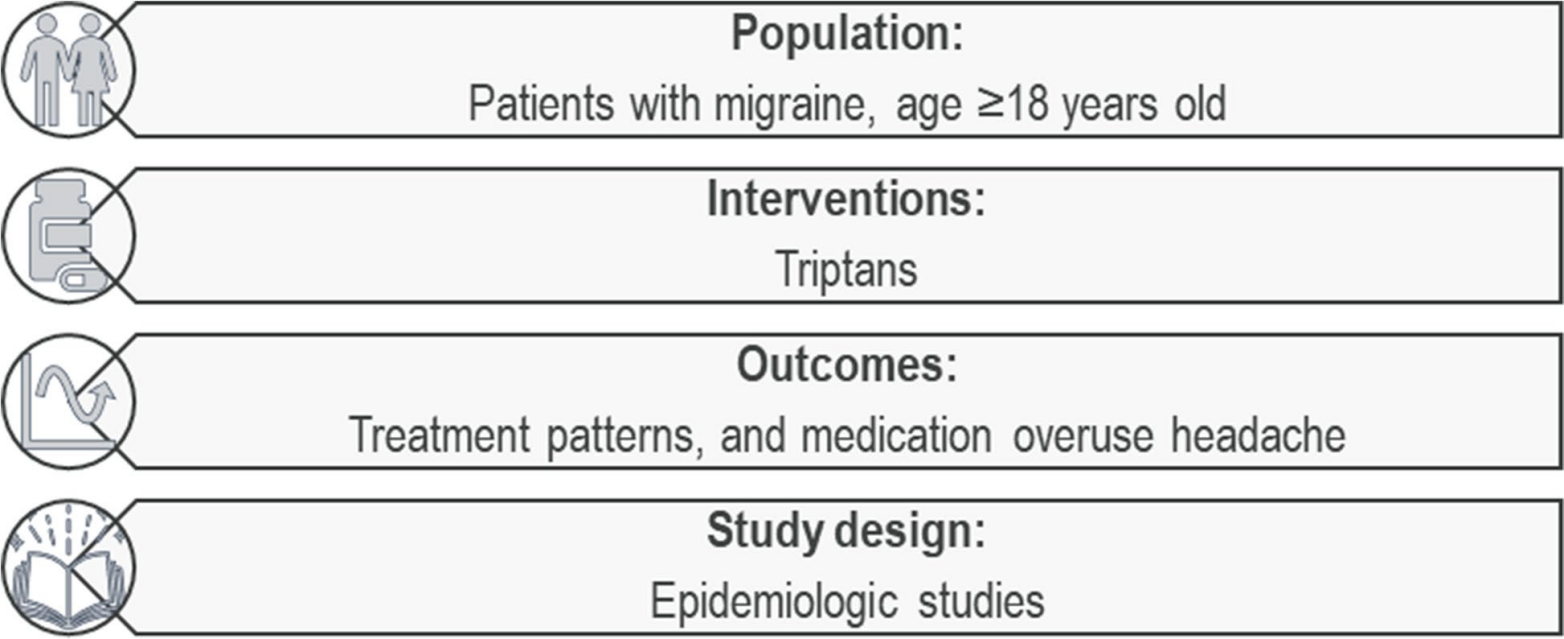
PICOS criteria

A SLR was conducted, using MEDLINE and EMBASE databases from database inception to July 2019, to identify articles that characterize (1) patterns of switching and discontinuation among triptan users, (2) triptan overuse among patients with MOH, and (3) describe the associated burden. The research questions described here (Fig. 1) were a subset of a broader SLR of migraine studies, and tables of the search terms and Population, Intervention, Comparators, Outcomes, Study Design (PICOS) criteria for the full SLR can be found in Suppl Tables 1 and 2.

The search strategy targeted studies of adults with migraine and included terms related to migraine and its treatment, as well as study design filters. Animal studies were excluded, and articles were limited to those published in English. Study screening and data extraction were performed in duplicate according to PRISMA guidelines [33]. Abstracts retrieved from the search strategy were independently screened by two reviewers, as were full-text articles identified for inclusion following abstract screening. Consensus was reached through discussion with a third party – under the guidance of the PICOS criteria – when reviewers initially disagreed on whether a record should be included.

Data were extracted by two reviewers; for continuous variables, the mean, median, standard deviation, and range were extracted whenever available. For dichotomous and categorical variables, the number of individuals and proportion were extracted. Study quality assessment was performed using the STROBE (Strengthening the Reporting of Observational Studies) criteria (Suppl Table 3) [34].

### Study and patient characteristics

Study characteristics extracted include the study author, year of publication, sampling frame, sample size, case ascertainment, and country. Patient characteristics for studies reporting on treatment patterns among triptan users included an indicator of incident vs. prevalent triptan users, and age and sex distribution within each study. For studies reporting on triptan overuse among patients with MOH, patient characteristics included the care setting and the treatment for MOH, age and sex distribution, percentage of the sample with MOH who had a history of migraine, and headache or migraine frequency (where available).

### Treatment patterns

**Fig. 2:**
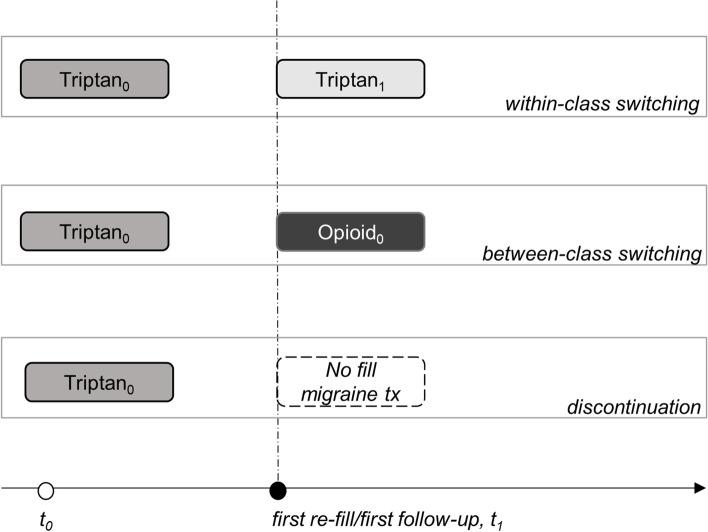
Discontinuation, within class switching, and between class switching

Patterns of switching and discontinuation among triptan users were described (Fig. 2). Rates of discontinuation were characterized by the percentage of patients that discontinued triptans where no fill for a different prescription triptan agent or class of migraine-specific or non-specific prescription therapies were observed (i.e. *discontinuation*). Two different forms of switching patterns were summarized: 1) switching to a different triptan agent regardless of the route of administration or dose (i.e. *within-class switching*, WCS), and 2) switching to a different class of migraine-specific or non-specific prescription therapies (e.g. opioid, NSAID; i.e. between-class switching, BCS). Both BCS overall and by acute medication agent were extracted to inform the frequency of BCS among triptan users and the distribution of subsequent therapies. The percentage of patients with BCS across the identified studies was plotted by acute medication agent on a bar plot. To further characterize triptan response rates, the percentage of the sample who refilled one or more prescriptions of their index triptan within 90 days before the end of the 1 or 2-year follow-up period (i.e. *one or two-year retention rate*) were extracted. Impacts on HRQoL associated with discontinuation, WCS, and BCS, as well as reasons for switching and discontinuing triptans were extracted where available, to characterize the unmet need among triptan users (both prevalent and incident users).

### Medication overuse headache

Acute medication use among patients with MOH was characterized by the percentage of patients with MOH that overused triptans, simple analgesics, combination analgesics, NSAIDS, ergots, or opioids; alone or in combination. The duration and frequency of medication overuse before MOH developed were also extracted. Rates of unsuccessful withdrawal (inability to discontinue the overused acute medication) after one-year were summarized, and reasons for unsuccessful withdrawal were extracted where available.

## Results

**Fig. 3:**
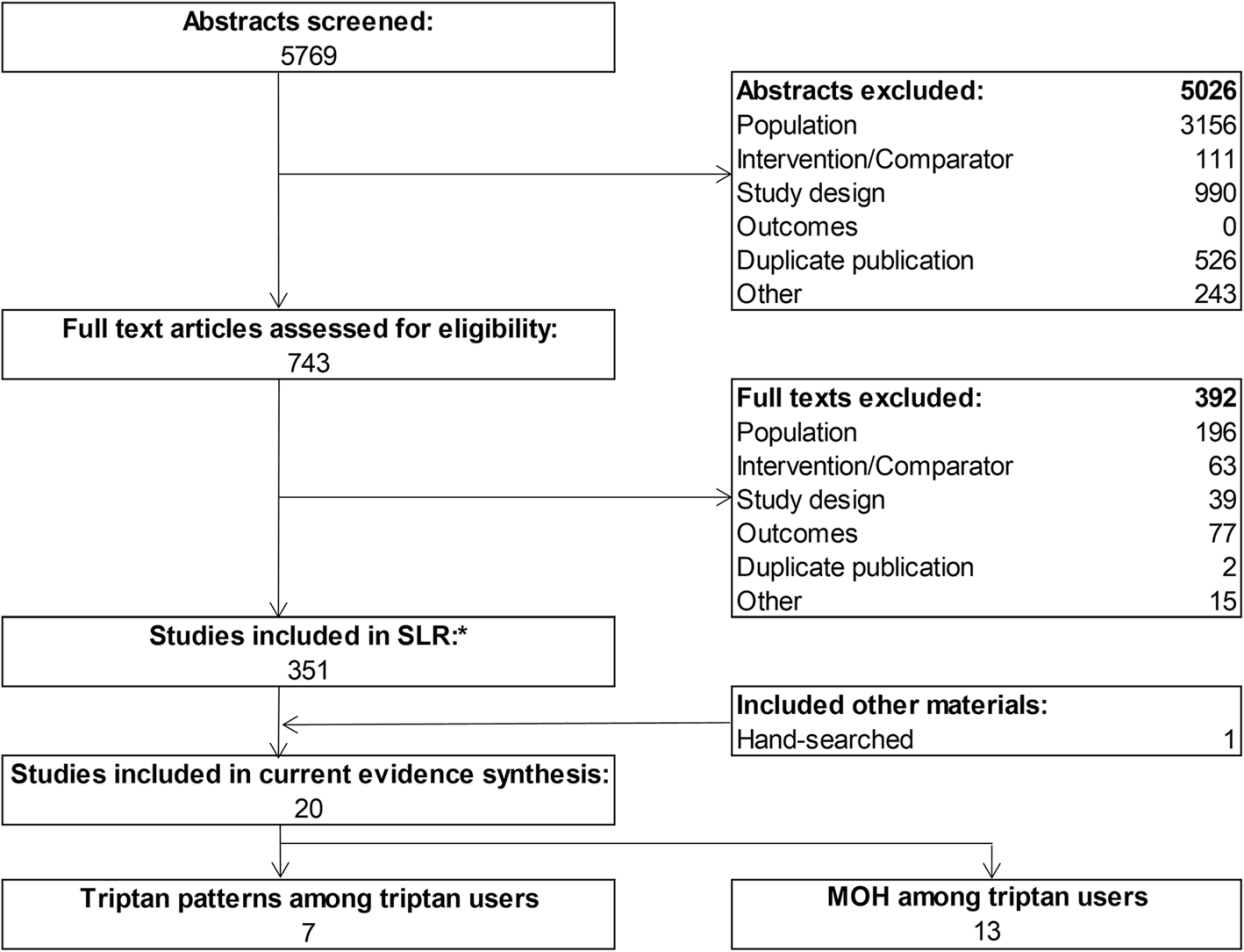
PRISMA flow diagram. Abbreviations: MOH = Medication overuse headache; SLR = systematic literature review. Note: *Methods to identify studies in the current study encompassed a broader focus (see Suppl tables 1 and 2)

From 5769 records identified, 743 full-text articles were screened, and 20 studies were identified that met the PICOS criteria in the current study (Fig. 1). The 20 studies included seven treatment patterns studies among triptans users [35–41] and 13 studies among patients with MOH (Fig. 3) [42–54]. Findings from the study quality assessment (based on STROBE guidelines) are shown in Suppl Table 4. Overall the quality of the included studies was fair as the articles included failed to comply with six to 20 of the 34 STROBE items. In general, the articles provided sufficient details when reporting the setting, sample size, and variables including potential confounders, as well as they thoroughly addressed key findings with reference to the study objective. However, sources of funding, explanations for how quantitative variables were handled in the analyses, methods for missing data, and efforts to assess bias were often sparsely described, if at all reported.

### Study and patient characteristics

Patient and study characteristics of the seven studies that reported on treatment patterns among triptan users are listed in Table 1 [35–41]. The identified studies included patients from North America [35, 38, 39, 41], Asia [36], and Europe [37, 40], and comprised four database studies [36, 38–40], one chart review [37], and two survey questionnaires [35, 41]. Patients were identified in database studies through International Classification of Diseases, 10th addition (ICD-10) codes for migraine (G43) or through medical records using ICHD criteria. Two surveys assessed triptan treatment patterns among prevalent or “current” triptan users (i.e. those who received triptans before the index date in the study) [35, 41], while the remaining studies included incident or “new” triptan users (i.e. those who did not receive triptans before the index date in the study) [36–40]. The mean age of the patients ranged from 37 to 47 years [37, 41], and the percentage of the sample that was female ranged from 73 to 89% [35, 41].

**Table 1:** Study and patient characteristics – switching and discontinuation patterns among triptan users

Citation	Country (Study period)	Study type	Study name/ database	Sample	Subgroup	n	Mean age, yrs	% female
Alam, 2018 [35]	US (2016)	Multicenter cross-sectional, Survey	MAST study	Current users	Overall	15,133	43.1	73.0
Chen, 2014 [36]	Taiwan (2005-2008)	Database study	NHIR Database	Incident users	Overall	13,951	41.3	77.0
Fischer, 2016 [37]	Austria (2009-2012)	Single center chart review	Outpatient clinic, U of Innsbruck	Incident users (first/new agent)	Overall	126	37.2	88.1
Katic, 2011 [38]	US (2001-2005)	Database study	i3 InVision Data Mart	Incident users	Overall	40,892	37.8	78.9
Lombard, 2018 [39]	US (2012-2014)	Database study	Truven MarketScan	Incident users	Overall	124,556	30.0-49.0	81.0
Ng-Mak, 2012 [40]	EU (2006-2008)	Database study	IMS Disease Analyzer	Incident users	UK	3618	41.3	75.6
					France	2051	38.9	75.8
					Germany	954	41.6	78.0
Serrano, 2013 [41]	US (2005-2009)	Multicenter prospective, Survey	AMPP study	Current users	Consistency group A	700	47.3	89.0
					Consistency group B	697	47.2	88.5
					Consistency group C	687	47.1	88.6

Consistency group A: Did not switch to another triptan in any couplet and consistently used the same triptan across years in their first eligible couplet; Consistency group B: Did not switch to an opioid/ barbiturate in any couplet and consistently used the same triptan across years in their first eligible couplet; Consistency group C: Did not switch to an NSAID in any couplet and consistently used the same triptan across years in their first eligible couplet

*Abbreviations*: *AMPP* American Migraine Prevalence and Prevention, *EU* Europe, *IMS* Information Management System, *MAST* Migraine in America Symptoms and Treatment, *NHIR* National Health Insurance Research, *SC* Subcutaneous inj., *U* University, *US* United States, *Yrs* Years

In Table 2, the patient and study characteristics for the 13 studies which described acute medication use among patients with MOH are summarized [42–54]. Among the included studies, four were chart review studies [45, 47, 49, 50], four were prospective cohort studies [48, 51, 53, 54], and five were questionnaire/surveys [42–44, 46, 52]. Most studies included patients from Europe [42, 44–46, 48, 49, 51–55], while the remaining studies included patients from North America [50], Asia [47], and South America [43]. Patients were identified through medical records using ICHD criteria. All patients were seen at either inpatient or outpatient centers, and most were treated via withdrawal of the acute medication [42, 45–49, 51–53], followed by a combination of withdrawal of the offending acute medication and initiation of migraine prophylactic therapy [44, 50], and lastly initiation of prophylaxis alone [54]. The percentage of patients with MOH who had a history of migraine ranged from 8 to 100% [43, 45, 46, 52]. The mean age of patients studied with MOH ranged from 38 to 50 years [44, 46, 53]; de Rijk et al. included patients with MOH who were older than 62 years, and their mean age was 70 years [45], Among patients with MOH, the percentage of the sample who were female ranged from 30 to 91% [46, 49], and headache frequency ranged from 17 to 27 days per month [52, 53].

**Table 2:** Study and patient characteristics – medication overuse headache among triptan users

Citation	Country (Study period)	Study type	Sample (MOH care setting, treatment)	n, MOH	% previous history migraine	Headache criteria	MHD	Mean age, yrs	% female
Benz et al., 2017 [42]	Switzerland (2012, 2014)	Single center cross-sectional, survey	Inpatient and outpatient, withdrawal of acute medication, prophylaxis	51	90.0	ICHD-II beta	NR	47.3	72.5
Chagas, 2015 [43]	Brazil (2009, 2010)	Single center prospective, survey	Outpatient, NR	29	100	ICHD-II	NR	44.2	82.8
Creac’h et al., 2009 [44]	France (2004, 2006)	Multicenter cross-sectional, survey	Inpatient, withdrawal of acute medication, prophylaxis; all overused triptans	163	99.0	ICHD-II	26.0	50.0	85.0
de Rijk et al., 2018 [45]	France (2006, 2015)	Single center, chart review	Inpatient, withdrawal of acute medication; > 65 years	79	100	ICHD-II beta	NR	69.5	79.1
Grande et al., 2011 [46]	Norway (2005, 2008)	Single center prospective, survey	Inpatient, education about medication overuse, prophylaxis	109	8.0	ICHD-II	22.0	37.5	30.3
Imai et al., 2007 [47]	Japan (NR)	Single center chart review	Inpatient and outpatient, withdrawal of acute medication	47	90.0	ICHD-II	NR	NR	NR
Katsarava et al., 2003^a^ [48]	Germany (NR)	Single center prospective cohort	Outpatient, withdrawal of acute medication	96	71.0	ICHD-I	NR	43.0	81.0
Kluonaitis et al., 2017 [49]	Lithuania (2015, 2016)	Single center chart review	Outpatient, likely withdrawal of acute medication, patients on prophylactic medication were excluded	87	67.0	ICHD-II beta	24.1	43.8	90.8
Lake et al., 2009 [50]	US and Canada (NR)	Single center chart review	Inpatient, withdrawal from acute mediation, prophylaxis, cognitive-behavioral therapy	158	85.0	ICHD-II	NR	40.0	79.4
Limmroth et al., 2002^a^ [51]	Germany (NR)	Single center prospective cohort	Outpatient, withdrawal of acute medication	96	71.0	ICHD-I	NR	43.0	81.0
Radat et al., 2005 [52]	France (NR)	Single center cross-sectional, survey	Inpatient, withdrawal of acute medication	41	100	ICHD-II	27.0	NR	NR
Zeeberg et al., 2006 [53]	Denmark (2002, 2003)	Single center prospective cohort	Outpatient, withdrawal of acute medication	216	53.0	ICHD-II	17.0	48.0	73.0
Zidverc-Trajkovic et al, 2007 [54]	Serbia (2000, 2005)	Single center prospective cohort	Inpatient, prophylaxis	240	67.1	ICHD-II	24.0	41.5	75.8

*Abbreviations*: *ICHD* The International Classification of Headache Disorders, *MOH* Medication overuse headache, *MHD* Monthly headache days, *NR* Not reported, *US* United States, *Yrs* Years

^a^Same patient population, different outcomes

### Treatment patterns

**Fig. 4:**
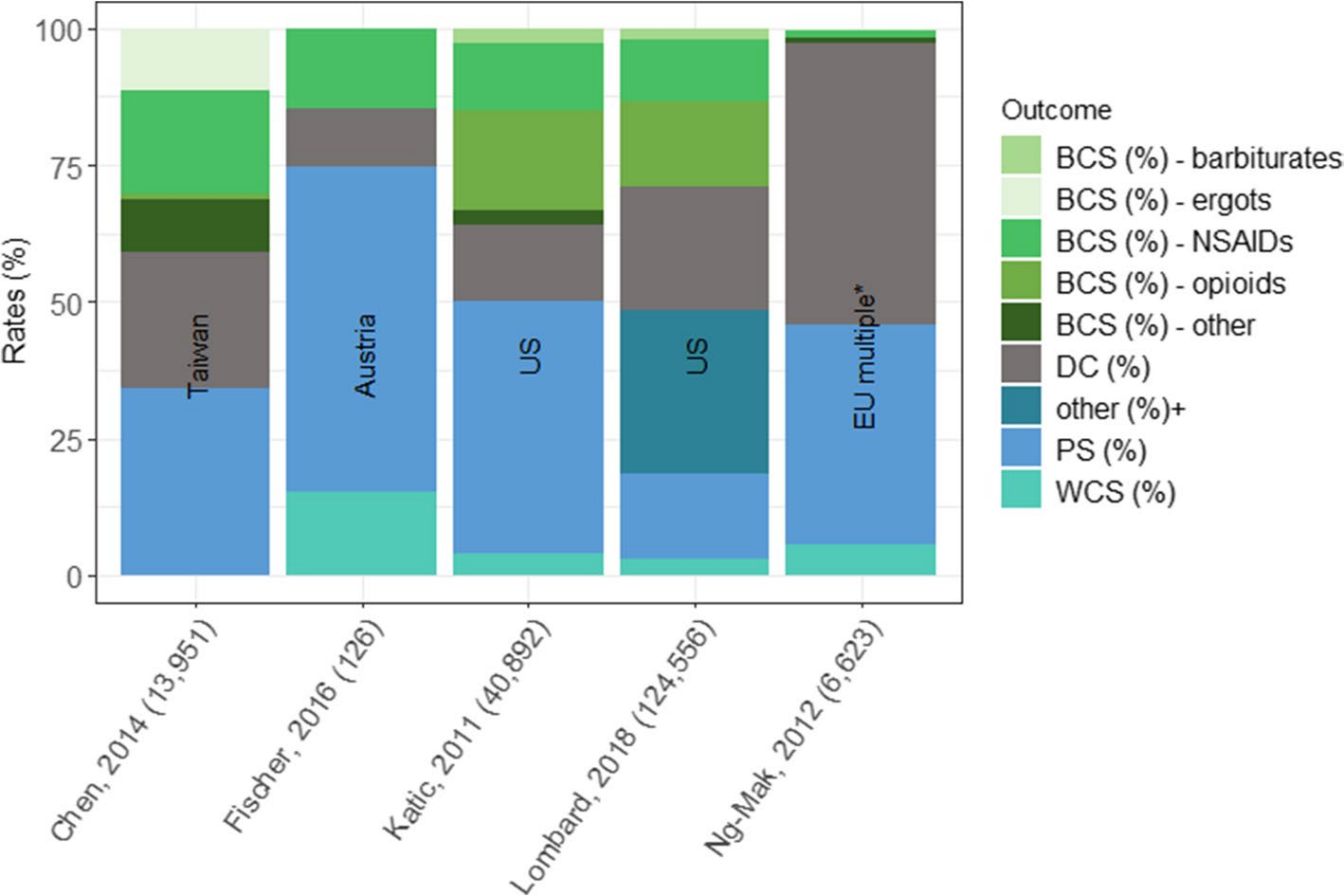
BCS, WCS and discontinuations by country and medication class. Abbreviations: BCS = between class switches; DC = discontinuers; EU = Europe; NSAID = nonsteroidal anti-inflammatory drug; PS = persistent users; WCS=Within class switches. Note: *Included UK, Germany, and France, +further details about switching patterns were only descried among patients with 1 switch during the follow-up period

The triptan agents and route of administration used (due to differences in health care systems and study period), as well as the timepoint at which the outcomes were assessed (due to differences in study design), varied across studies reporting on rates of triptan switching and discontinuation (Table 3) [36–40]. Rates of discontinuation ranged from 11% at first follow-up visit among an Austrian sample [37] to 55% at first refill among French and German samples (Table 3 and Fig. 4) [40]. Rates of WCS ranged from < 1% at first refill among a Taiwanese sample [36] to 15% at first follow-up visit among an Austrian sample [37]. Rates of BCS ranged from 2% at first refill among British and German samples [40] to 41% at first refill among a Taiwanese sample (Table 3 and Fig. 4) [36]. Among the overall cohort, the percentage with BCS to an opioid ranged from 0% (at first refill; France and Germany) [40] to 18.2% (at first refill; US) [38] and to a barbiturate ranged from 0% (at first refill; UK, France, Germany [40]; Taiwan) [36] to 2.6% (at first refill; US) (Table 3 and Fig. 4) [38]. The rate of one-year triptan retention was reported in one study among a US sample at 15% [39] and two-year retention rates ranged from 4% (Taiwan) [36] to 13% (UK) (Table 3 and Fig. 4) [40].

**Table 3:** Switching and discontinuation patterns among triptan users

Citation	Country	Triptans (forms)	n	PS (%)	Time point for DCS, WCS, and BCS	DC (%)	WCS (%)	BCS (% of total cohort)				
								Overall	Opioid	NSAIDs^**ab**^	BAR	Ergots
Chen, 2014 [36]	Taiwan	suma, riza (oral, sc)	13,951	4.0 by 2-yrs	1st refill	24.8	0.01	40.9	1.2	18.8	0	11.3
Fischer, 2016 [37]	Austria	ele, frova, suma, zolmi (NR)	126	NR	1st follow-up visit	10.8	15.1	14.6^a^	NA^a^	14.6^ab^	NR	NR
Katic, 2011 [38]	US	almo, nara, ele, frova, suma, riza, zolmi (all)	40,892	6.4 by 2-yrs	1st refill	13.7	4.0	36.2	18.2	12.5	2.6	0
Lombard, 2018 [39]^c^	US	NR	124,556	15.0 by 1-yr	with one change after 1-year	22.5	3.2	29.1	15.5	11.6^a^	1.9	NR
Ng-Mak, 2012 [40]	UK	almo, ele, frova, nara, riza, suma, zolmi (oral)	3618	13.0 by 2-yr	1st refill	48.5	4.9	2.3	0.1	0.9	0	0.2
	France		2051	6.0 by 2-yr	1st refill	54.9	6.8	4.0	0	2	0	0.6
	Germany		954	9.0 by 2-yr	1st refill	54.7	6.3	2.3	0	1.3	0	0.1

*Abbreviations*: *BAR* Barbiturates, *BCS* Between class switches, *DC* Discontinuers, *EU* Europe, *NR* Not reported, *PS* Persistent users, *SC* Subcutaneous inj, *US* United States, *WCS* Within class switches, *yr(s)* Year(s)

^a^Acute medication other than triptans included nonsteroidal anti-inflammatory drugs and paracetamol. Opioids are not used in clinical routine for treatment of migraine headache in Austria

^b^NSAIDs/acetaminophen

^c^In Lombard et al. there were 30% of patients who had 2+ switched, and not further information was given to characterize switching among these patients

Two studies described the burden associated with switching and discontinuation of triptans over time [37, 41]. Among current triptan users, change in 3-month headache-related disability was assessed from the first to second year of follow-up [41]. Compared to those who retained the same triptan agent at follow-up, neither WCS nor BCS were associated with significant improvements in headache-related disability across headache frequency strata, and among patients with more frequent migraines (10 or more headache days per month) BCS to NSAIDS was associated with significant increases in headache-related disability [41]. To the best of our knowledge, the study authors did not report the number of times patients were switched to different triptans [41]. Similarly, in Fischer et al., among patients who were treated with a new triptan agent or their first triptan, significantly higher headache-related impact scores were observed in those who had not refilled their initially prescribed triptan compared to those who had retained the same triptan at the first follow-up visit (*P* < .029) [37]. In three of the included studies, reasons for WCS, BCS, and discontinuation were described [35–37]. Commonly reported reasons for WCS were insufficient treatment response or adverse events, reasons for BCS included contraindications for triptan use, and reasons for discontinuation from therapy included attack-freedom [35–37].

### Medication overuse headache

Rates of acute medication use among patients with MOH are shown in Table 4. The percentage overusing triptans varied from 2% among a Japanese sample [47] to 46% among a French sample [52]. The percentage overusing opioids ranged from 0% among Japanese [47] and German samples [49] to 48% among a North American sample [50]. The percentage overusing ergots varied from 2% among Japanese [47] and German samples [49] to 43% among a Serbian sample [54] (Table 4). Among the sample from North America, who attended an inpatient program, opioids (48%) and triptans (16%) were the most commonly overused medications [50]. Creac’h et al. (France, cross-sectional survey, 2004 to 2006), included patients with MOH who were all triptan overusers; approximately half of the patients with MOH only overused triptans and the other half overused triptans in combination with another agent (e.g. triptans + combination analgesics [36%], triptans + opioids [60%], triptans + ergots [0.6%]) [44].

**Table 4:** Medication overuse among triptan users with medication overuse headache

Citation	n, MOH	Medication overuse by class (%)						
		Triptans	Analgesics	Simple analgesics	NSAIDS	Combination analgesics	Ergots	Opioids
Benz et al., 2017 [42]	51	14.0	NR	NR	NR	24.0	NR	2.0
Chagas, 2015 [43]	29	14.0	NR	NR	28.0	NR	NR	NR
Creac’h et al., 2009 [44]	163	100	NR	42.0	NR	67.0	0.6	60.0
de Rijk et al., 2018 [45]	79	31.7	NR	38.0	17.7	70.9	8.9	2.5
Grande et al., 2011 [46]	109	6.0	NR	73.0	NR	19.0	NR	NR
Imai et al., 2007 [47]	47	2.1	8.5	NR	NR	85.1	2.1	0.0
Katsarava et al., 2003^a^ [48]	96	39.0	48.0	NR	NR	NR	13.0	NR
Kluonaitis et al., 2017 [49]	87	41.8	NR	38.8	27.3	38.8	1.5	16.1
Lake et al., 2009 [50]	158	16.0	NR	NR	NR	NR	NR	48.0
Limmroth et al., 2002^a^ [51]	96	39.0	48.0	NR	NR	NR	13.0	7.0
Radat et al., 2005 [52]	41	46.3	NR	NR	29.3	NR	22.0	NR
Zeeberg et al., 2006 [53]	216	20.0	NR	29.0	NR	42.0	4.0	6.0
Zidverc-Trajkovic etr al, 2007 [54]	240	18.9	70.8	NR	25.8	21.7	42.8	6.0

*Abbreviations*: *MOH* Medication overuse headache, *NR* Not reported, *NSAID* Nonsteroidal anti-inflammatory drug

^a^Same patient population, different outcomes

One study, a prospective study of patient with MOH in Germany, reported on the duration and frequency of medication use prior to the diagnosis of MOH [51]. Limmroth et al. found that overuse of triptans lead to more rapid onset of MOH (1.7 years) than ergots (2.7 years) and analgesics (4.8 years; including combination, simple, and opioids analgesics) [51]. Compared to analgesics use as a whole, overuse of opioids led to more rapid onset of MOH (2.2 years).

After one-year, the percentage of patients with MOH who were unsuccessful in withdrawing from their overused medication ranged from 3% (Serbia, prospective cohort study, inpatient setting with prophylaxis, 2000 to 2005) [54] to 19% (Germany, prospective cohort study, outpatient withdrawal of acute medication, study period NR) [48]. Medication agent [48, 53], increased headache frequency (*p* = .008) [54], and increased disability (*p* = .045) [54] were associated with unsuccessful withdrawal; patients overusing triptans were more successful at withdrawal than those overusing analgesics overall (*P* < .0001 - ≤ .002), and specifically opioids, or combination analgesics (*P* < .0001) [48, 53].

## Discussion

In the identified studies describing treatment patterns, rates of WCS were low, and many new triptan users switched from their index triptan to another class of medication at their first refill; most notably high rates of BCS to opioids were observed among US populations compared to non-US/other populations [38, 39]. Among the US samples identified in this review, 18.2% at first refill or 15.5% after the first year switched from their index triptan to an opioid [38, 39]. Compared to consistent treatment with the same triptan after 1 year, WCS was not associated with improvement in headache-related disability and BCS to certain acute medication classes was associated with increases in headache-related disability [41]. High rates of switching to non-specific acute medications and low two-year retention rates demonstrate a lack of tolerable triptan options. Among patients with MOH treated at inpatient and outpatient clinics, withdrawal was more difficult among patients with increased headache frequency, increased disability, or among those over using opioids or combination analgesics; highlighting that for more severe patients the treatment of MOH can be complex.

Strengths of this review include the use of rigorous systematic literature review methods to identify studies of real-world triptan use among patients with migraine and acute medication use among patients with MOH; including those that focused on precisely estimating treatment patterns among incident triptan users as well as studies reporting on various outcomes among patients with MOH. The following knowledge gaps in the literature were identified: (1) rates of WCS, with respect to the triptan dosage or route of administration, (2) headache-related disability among patients who switch to a different triptan agents by the number of switches, and (3) the clinical, humanistic, and economic burden among triptan users that develop MOH.

As with all evidence syntheses, this SLR was limited by the quality, validity, heterogeneity, and reporting accuracy of the included studies. Using the STROBE quality assessment tool we deemed the quality of the identified observational studies to be fair. Importantly, this quality assessment highlighted that only one study identified here described any efforts to address potential sources of bias. Variability in health care settings by country, study designs, data sources, patient populations, and how outcomes were defined across studies contributed to heterogeneity across estimates.

The variability in health care settings by country had many implications across the range of outcomes reported throughout this SLR. First, opioid use is more common in the US compared to in Europe as opioids are still widely used in the US to treat severe attacks in emergency departments [9, 10, 49]. Though this review only identified treatment patterns studies with relatively short follow-up it is important to understand the risk of opioid dependence among triptan non-responders, as other studies have suggested that opioid dependence among patients not adequately managed on triptans constitutes a significant public-health concern [56, 57]. Secondly, in certain countries triptans are available OTC (e.g. Japan and Germany) [40, 47]; therefore, not all triptan use would be captured in claims databases analysis, which contribute to underestimation of WCS rates. Furthermore, the timing of triptan uptake, overall and by agent and route of administration, varies by country and study period and thus was not consistent across the included studies. For example, in Imai et al. triptans were only overused by 2% of the sample; however, authors noted that triptans only recently entered the market in Japan at the time of study. Lastly, many patients manage their migraines with multiple acute medications at a time. Therefore, describing rates of acute medication use and success of withdrawal by medication agent is challenging. Two included studies described the differences in the clinical burden associated with MOH secondary to the use of triptans alone versus other treatments alone or in combination with triptans [44, 51]. Creac’h et al. noted that compared to patients with MOH who were purely overusing triptans, patients overusing triptans in combination experience more frequent and severe migraine, and were characterized by stronger dependence on acute treatments of headaches [44]. Authors suggested that these patients may require a more intensive prophylactic therapy and specific behavioral management [44, 51].

Rates of switching and discontinuation among triptan users and rates of acute medication use among patients with MOH were characterized descriptively; because of the heterogeneity of patients across the included studies, no formal meta-analysis was performed. The mean age and the percentage of the sample who were female varied across the included studies. The headache classification used varied by study design (ICD in database studies vs. ICHD in cohort studies), by country, and over time. Specifically, the version of ICHD criteria for MOH used varied by study period; ICHD-II criteria was used in most studies. Furthermore, the headache frequency was infrequently reported, and the percentage of patients with previous migraine ranged from 8% [46] to 100% [43, 45, 52], Together these strongly affected the difference in rates of acute medication use by agent among patients with MOH. It is also important to note, that we required studies of confirmed MOH. Therefore, studies of patients with chronic migraine overusing acute medications were not described here, which may have led to identifying a more severe set of patients with MOH seen at inpatient and outpatient clinics. A future SLR describing rates of acute medication use among patients with choric migraine as well as patients with MOH is warranted.

## Conclusion

Under current AHS guidelines, patients must either have contraindications to the use of triptans or have failed to respond to or tolerate at least two oral triptans to be considered for treatment with novel therapies [8, 23]. The evidence summarized here highlights that rates of WCS are low and many patients turn to other acute medication at their first refill. Patients may experience no improvement in disability when switching from one triptan agent to another, or experience increasing disability and/or increasing migraine frequency when turning to traditional acute treatment for migraine. Variability in health care settings, patient severity, and study design contributed to the heterogeneity across the identified studies.

## Supplementary Information

**Additional file 1: Suppl Table 1.** Search Strategy. **Suppl Table 2.** Systematic Review Selection Criteria. **Suppl Table 3.** STROBE statement recommendation checklist. **Suppl Table 4.** Quality assessment using STROBE.

## Data Availability

All data presented in this systematic review are derived from published studies and are available from the first author on reasonable request.

## Abbreviations

SLR: Systematic literature review
NSAIDS: Nonsteroidal anti-inflammatory drug
CV: Cardiovascular
HRQoL: Health-related quality of life
WCS: Within-class switching
BCS: Between-class switching
MOH: Medication overuse headache
AHS: America Headache Society
PICOS: Population, Intervention, Comparators, Outcomes Study Design
STROBE: Strengthening the reporting of observational studies
ICD-10: International Classification of Diseases, 10th addition
ICHD: The International Classification of Headache Disorders
NR: Not reported
OTC: Over the counter
AMPP: American Migraine Prevalence and Prevention
EU: Europe
IMS: Information Management System
MAST: Migraine in America Symptoms and Treatment
NHIR: National Health Insurance Research
SC: Subcutaneous inj
US: United States
U: University
MHD: Monthly headache days
Yr: Year
DC: Discontinuers
BAR: Barbiturates
