# Supplementary material for: The burden of medication overuse headache and patterns of switching and discontinuation among triptan users: a systematic literature review
Source: BMC Neurol. 2021 Nov 2;21:425. doi: 10.1186/s12883-021-02451-x (PMC8561931; doi:10.1186/s12883-021-02451-x)
Supplement: Supplementary file 1 — Additional file 1: Suppl Table 1. Search Strategy. Suppl Table 2. Systematic Review Selection Criteria. Suppl Table 3. STROBE statement recommendation checklist. Suppl Table 4. Quality assessment using STROBE. [file 12883_2021_2451_MOESM1_ESM.pdf]

Supplemental materials

Title: The burden of medication overuse headache and patterns of switching and discontinuation among triptan users: A systematic literature review

Authors: Alison M Deighton, BASc[1]; Linda A Harris, MPH[2]; Karissa Johnston, PhD[1]; Shomari Hogan, MD;[2] Lynn A Quaranta, PharmD;[2] Gilbert L'Italien, PhD;[2] Vlad Coric, MD[2]

[1]Broadstreet HEOR, 201-343 Railway St, Vancouver BC, Canada
[2]Biohaven Pharmaceuticals, 215 Church Street, New Haven CT, USA

Suppl Table 1. Search Strategy

Topic	Order	Search term	Results
MEDLINE - Population	1	exp Migraine Disorders/	85,394
	2	(migrain* or headache* or cephalgi* or cephalalgi*).ti,ab.	244,906
	3	1 or 2	260,002
MEDLINE - Treatments	4	exp Serotonin 5-HT1 Receptor Agonists/	6,903
	5	(triptan* or tryptamine*).mp.	21,355
	6	exp sumatriptan/	10,936
	7	(sumatriptan or treximet or Imitrex or Imigran or Alsuma or Adracon or Altaxa or Apigrane or Cinie or Frimig or Illument or Imigen or Imigrane or Migralgin or Migraneitor or Nazdav or Sapphirex or Sumatran or Sumax or Suminat or Youshu).mp.	12,769
	8	(zolmitriptan or zomig or flezol or zomigoro or AscoTop).mp.	3,491
	9	(rizatriptan or Maxalt).mp.	2,884
	10	(eletriptan or relpax).mp.	1,696
	11	(naratriptan or Amerge or Naramig or Colatan).mp.	2,017
	12	(frovatriptan or Frova or Frovelan or Allegro).mp.	2,016
	13	(almotriptan or axert or almogran).mp.	1,490
	14	exp ergotamine/	7,004
	15	exp dihydroergotamine/	6,413
	16	(Ergomar or Cafergot or Ergotamin* or Gynergen or Ergostat or dihydroergotamine or ergot*).mp.	27,292
	17	exp Anti-Inflammatory Agents, Non-Steroidal/	903,879
	18	exp ibuprofen/	56,471
	19	exp aspirin/	246,328
	20	(ibuprofen or motrin or advil).mp.	64,091
	21	excedrin.mp.	362
	22	(NURTEC ODT or BMS-927711).mp.	103
	23	(REYVOW or COL-144 or LY573144).mp.	165
	24	(UBRELVY or MK-1602).mp.	85
	25	NXN-188.mp.	30
	26	or/4-25	962,130
MEDLINE - RCTs	27	(randomized controlled trial or controlled clinical trial).pt.	574,119
	28	(randomi#ed or randomly).ti,ab.	1,987,037
	29	placebo.ab.	482,595
	30	clinical trials as topic.sh.	187,705
	31	trial.ti.	477,429
MEDLINE - Observational studies	32	exp case-control studies/ or case?control stud*.mp.	1,167,766
	33	exp cohort studies/ or cohort stud*.mp.	2,488,618
	34	exp cross-sectional studies/ or cross?sectional stud*.mp.	608,762

Topic	Order	Search term	Results
	35	exp observational study/ or exp prospective studies/ or exp retrospective studies/ or (observ* stud* or prospective stud* or retrospective stud*).mp.	2,995,567
	36	exp epidemiologic studies/ or epidemiologic* stud*.mp.	5,627,577
	37	exp follow-up studies/ or follow?up stud*.mp.	2,046,104
	38	exp longitudinal studies/ or longitudinal stud*.mp.	320,971
MEDLINE - Included study designs	39	or/27-38	10,002,615
	40	letter/	2,058,565
	41	editorial/	1,113,206
	42	news/	196,084
	43	exp historical article/	389,523
	44	anecdotes as topic/	44,017
	45	comment/	786,317
	46	(letter or comment*).ti.	328,955
	47	animals/ not humans/	5,525,499
MEDLINE - Excluded study designs and publication types	48	or/40-47	9,585,759
	49	3 and 26 and 39	11,012
	50	49 not 48	10,737
	51	50 use ppezv	2,593
Embase - Population	52	exp Migraine/	85,394
	53	(migrain* or headache* or cephalgi* or cephalalgi*).ti,ab.	244,906
	54	52 or 53	260,002
Embase - treatments	55	exp serotonin 1 agonist/	1,269
	56	exp triptan derivative/	3,331
	57	(triptan* or tryptamine*).mp.	21,355
	58	exp sumatriptan/	10,936
	59	exp naproxen plus sumatriptan succinate/	42
	60	(sumatriptan or treximet or Imitrex or Imigran or Alsuma or Adracon or Altaxa or Apigrane or Cinie or Frimig or Illument or Imigen or Imigrane or Migralgin or Migraneitor or Nazdav or Sapphirex or Sumatran or Sumax or Suminat or Youshu).mp.	12,769
	61	exp zolmitriptan/	2,822
	62	(zolmitriptan or zomig or flezol or zomigoro or AscoTop).mp.	3,491
	63	exp rizatriptan/	2,337
	64	(rizatriptan or Maxalt).mp.	2,884
	65	exp eletriptan/	1,378
	66	(eletriptan or relpax).mp.	1,696
	67	exp naratriptan/	1,657
	68	(naratriptan or Amerge or Naramig or Colatan).mp.	2,017

Topic	Order	Search term	Results
	69	exp frovatriptan/	978
	70	(frovatriptan or Frova or Frovelan or Allegro).mp.	2,016
	71	exp almotriptan/	1,186
	72	(almotriptan or axert or almogran).mp.	1,490
	73	exp ergot derivative/	882
	74	exp ergotamine/	7,004
	75	exp dihydroergotamine/	6,413
	76	(Ergomar or Cafergot or Ergotamin* or Gynergen or Ergostat or dihydroergotamine or ergot*).mp.	27,292
	77	exp nonsteroid antiinflammatory agent/	704,982
	78	exp ibuprofen/	56,471
	79	exp acetylsalicylic acid/	246,328
	80	(ibuprofen or motrin or advil).mp.	64,091
	81	exp acetylsalicylic acid plus caffeine plus paracetamol/	8
	82	excedrin.mp.	362
	83	exp NURTEC ODT/	61
	84	(NURTEC ODT or BMS-927711).mp.	103
	85	exp REYVOW/	108
	86	(REYVOW or COL-144 or LY573144).mp.	165
	87	exp UBRELVY/	62
	88	(UBRELVY or MK-1602).mp.	85
	89	NXN-188.mp.	30
	90	or/55-89	813,464
Embase - RCTs	91	random*.ti,ab.	2,494,896
	92	factorial*.ti,ab.	63,624
	93	(crossover* or cross?over*).ti,ab.	132,251
	94	((doubl* or singl*) adj blind*).ti,ab.	382,155
	95	(assign* or allocat* or volunteer* or placebo*).ti,ab.	1,723,352
	96	crossover procedure/	59,948
	97	double blind procedure/	162,979
	98	single blind procedure/	35,862
	99	randomized controlled trial/	1,045,475
Embase - Observational studies	100	exp case control study/ or exp cohort analysis/ or exp cross-sectional study/ or exp observational study/ or (epidemiolog* stud* or case?control stud* or cohort stud* or cohort analys* or cross?sectional stud* or observ* stud* or follow?up stud* or longitudinal or prospective or retrospective).mp.	5,894,695
Embase - Included study designs	101	or/91-100	8,955,506
	102	letter.pt. or letter/	2,116,537
	103	note.pt.	763,447
	104	editorial.pt.	1,121,846
	105	(letter or comment*).ti.	328,955
	106	animal/ not human/	5,609,523

Topic	Order	Search term	Results
Embase - Excluded study designs and publication types	107	or/102-106	9,679,892
	108	54 and 90 and 101	8,194
	109	108 not 107	8,021
	110	109 use oemezd	5,982
Combined	111	51 or 110	8,575
	112	limit 111 to english language	8,088
	113	limit 112 to human$	7,583
	114	limit 113 to "review articles" [Limit not valid in Embase; records were retained]	**5,769**

Database searched: Embase/Medline

Search date: July 22, 2019

Suppl Table 2. Systematic Review Selection Criteria

**Population**	Acute migraine, both sexes, age ≥18 years old<br>Subject has at least 1-year history of migraine (with or without aura)
**Interventions/comparators**	Triptans, including:<br>• Sumatriptan (DFN-11, DFN-02, NP101)<br>• Treximet<br>• Almotriptan<br>• Eletriptan<br>• Frovatriptan<br>• Naratriptan<br>• Rizatriptan<br>• Zolmitriptan<br>Ergots, including:<br>• Ergotamine<br>• Dihydroergotamine (migranal)<br>Other targeted therapies:<br>• NURTEC(TM) ODT<br>• REYVOW™<br>• UBRELVY™ (MK-1602)<br>• NXN-188<br>Over the counter NSAIDs:<br>• Aspirin<br>• Advil<br>• Excedrin<br>Any intervention
**Outcomes**	Clinical outcomes<br>Treatment efficacy:<br>• Pain freedom<br>• Sustained Pain Freedom<br>• Pain relief<br>• Sustained Pain Relief<br>• Freedom from functional disability<br>• Freedom from most bothersome symptom (MBS)<br>• Probability of Requiring Rescue Medication<br>• Subjects self-report "normal" on the functional disability scale<br>• Freedom from Photophobia<br>• Freedom from Phonophobia<br>• Freedom from Nausea

	• Pain relapse<br>• Liver function tests (ALT/AST)<br>Treatment safety:<br>• Adverse events (AEs)<br>• Serious adverse events (SAEs)<br>• Nausea, vomiting<br>• Dizziness<br>• Fatigue/ malaise/somnolence<br>• Sensory disturbance<br>• Cardiovascular/cerebrovascular outcomes<br>• Chest pain<br>• Medication overuse headache<br>• Return to function<br>Subgroups<br>• Triptan refractory population<br>Epidemiology outcomes<br>Population characteristics<br>• Age<br>• Sex<br>• Region<br>• Comorbidities (including cardiovascular disease)<br>Clinical outcomes<br>• Treatment safety<br>• Cardiovascular/cerebrovascular outcomes<br>• Medication overuse headache<br>• Treatment patterns<br>• Return to function<br>• Risk of chronic migraine<br>○ Inadequate acute treatment and onset of chronic migraines<br>• Headache frequency<br>Economic outcomes<br>• Direct medical costs<br>• Indirect costs and lost work productivity<br>○ Days missed from work<br>○ Work-related disability<br>Quality of life outcomes<br>• HRQoL<br>○ PRO<br>○ Utility
**Study design**	Randomized controlled trials<br>Epidemiologic (prospective or retrospective) studies

Suppl Table 3. STROBE statement recommendation checklist

Section	Item No	Recommendation
Title and abstract	1	(a) Indicate the study's design with a commonly used term in the title or the abstract<br>(b) Provide in the abstract an informative and balanced summary of what was done and what was found
Introduction		
Background	2	Explain the scientific background and rationale for the investigation being reported
Objectives	3	State specific objectives, including any pre-specified hypotheses
Methods		
Study design	4	Present key elements of study design early in the paper

Section	Item No	Recommendation
Setting	5	Describe the setting, locations, and relevant dates, including periods of recruitment,<br>exposure, follow-up, and data collection
Participants	6	(a) *Cohort study*—Give the eligibility criteria, and the sources and methods of<br>selection of participants. Describe methods of follow-up<br>*Case-control study*—Give the eligibility criteria, and the sources and methods of<br>case ascertainment and control selection. Give the rationale for the choice of cases<br>and controls<br>*Cross-sectional study*—Give the eligibility criteria, and the sources and methods of<br>selection of participants<br><br>(b) *Cohort study*—For matched studies, give matching criteria and number of<br>exposed and unexposed<br>*Case-control study*—For matched studies, give matching criteria and the number of<br>controls per case
Variables	7	Clearly define all outcomes, exposures, predictors, potential confounders, and effect<br>modifiers. Give diagnostic criteria, if applicable
Data sources/measurement	8	For each variable of interest, give sources of data and details of methods of<br>assessment (measurement). Describe comparability of assessment methods if there<br>is more than one group
Bias	9	Describe any efforts to address potential sources of bias
Study size	10	Explain how the study size was arrived at
Quantitative variables	11	Explain how quantitative variables were handled in the analyses. If applicable, describe which groupings were chosen and why
Statistical methods	12	(a) Describe all statistical methods, including those used to control for confounding<br>(b) Describe any methods used to examine subgroups and interactions<br>(c) Explain how missing data were addressed<br>(d) *Cohort study*—If applicable, explain how loss to follow-up was addressed<br>*Case-control study*—If applicable, explain how matching of cases and controls was<br>addressed<br>*Cross-sectional study*—If applicable, describe analytical methods taking account of<br>sampling strategy<br>(e) Describe any sensitivity analyses
Results		
Participants	13	(a) Report numbers of individuals at each stage of study—e.g. numbers potentially eligible,

Section	Item No	Recommendation
		examined for eligibility, confirmed eligible, included in the study, completing follow-up, and<br>analyzed<br>(b) Give reasons for non-participation at each stage<br>(c) Consider use of a flow diagram
Descriptive data	14	(a) Give characteristics of study participants (e.g. demographic, clinical, social) and information<br>on exposures and potential confounders<br>(b) Indicate number of participants with missing data for each variable of interest<br>(c) Cohort study—Summarize follow-up time (e.g., average and total amount)
Outcome data	15	*Cohort study*—Report numbers of outcome events or summary measures over time<br>*Case-control study*—Report numbers in each exposure category, or summary measures of<br>exposure<br>*Cross-sectional study*—Report numbers of outcome events or summary measures
Main results	16	(a) Give unadjusted estimates and, if applicable, confounder-adjusted estimates and their<br>precision (e.g., 95% confidence interval). Make clear which confounders were adjusted for and<br>why they were included<br>(b) Report category boundaries when continuous variables were categorized<br>(c) If relevant, consider translating estimates of relative risk into absolute risk for a meaningful<br>time period
Other analyses	17	Report other analyses done—e.g. analyses of subgroups and interactions, and sensitivity<br>Analyses
Discussion		
Key results	18	Summarize key results with reference to study objective
Limitations	19	Discuss limitations of the study, taking into account sources of potential bias or imprecision.<br>Discuss both direction and magnitude of any potential bias
Interpretation	20	Give a cautious overall interpretation of results considering objectives, limitations, multiplicity<br>of analyses, results from similar studies, and other relevant evidence
Generalizability	21	Discuss the generalizability (external validity) of the study results
Other Information		
Funding	22	Give the source of funding and the role of the funders for the present study and, if applicable, for the original study on which the present article is based

Suppl Table 4. Quality assessment using STROBE

	STROBE Item Number																	
**Citation**	**1a**	**1b**	**2**	**3**	**4**	**5**	**6a**	**6b**	**7**	**8**	**9**	**10**	**11**	**12a**	**12b**	**12c**	**12d**	**12e**
Articles reporting on treatment patterns among triptan users																		
Chen, 2014(36)	Y	Y	Y	Y	Y	Y	Y	--	Y	Y	N	Y	N	Y	N	N	N	N
Lombard, 2018(39)*	Y	Y																
Ng-Mak, 2012(40)	Y	Y	Y	Y	Y	Y	Y	--	Y	Y	N	Y	N	Y	N	N	N	N
Alam, 2018(35)*	N	Y																
Fischer, 2016(37)	Y	Y	Y	Y	Y	Y	Y	--	Y	Y	N	Y	Y	Y	Y	N	N	N
Serrano, 2013(41)	N	Y	Y	Y	Y	Y	Y	--	Y	Y	N	Y	Y	Y	Y	N	N	N
Katic, 2011(38)	Y	Y	Y	Y	Y	Y	Y	--	Y	Y	N	Y	N	Y	Y	N	N	N
Articles reporting on MOH among triptan users																		
Katsarava et al, 2003(48)	Y	Y	Y	Y	Y	Y	Y	--	N	Y	N	Y	N	Y	Y	N	N	N
Kluo--itis et al, 2017(49)	Y	Y	Y	Y	Y	Y	Y	--	Y	Y	N	Y	--	Y	Y	N	N	N
Limmroth et al, 2002(51)	Y	Y	Y	Y	Y	Y	Y	--	Y	Y	N	Y	--	Y	Y	N	N	N
Zeeberg et al, 2006(53)	N	Y	Y	Y	N	Y	Y	--	Y	Y	Y	Y	--	Y	Y	N	N	N
Benz et al, 2017(42)	Y	Y	Y	Y	Y	Y	Y	--	Y	Y	N	Y	Y	Y	Y	Y	N	N
Creac'h et al, 2009(44)	Y	Y	Y	Y	Y	Y	Y	--	Y	Y	N	Y	--	Y	Y	N	N	N
de Rijk et al, 2018(45)	N	Y	Y	Y	Y	Y	Y	--	Y	Y	N	Y	N	Y	Y	N	Y	N
Grande et al, 2011(46)	Y	Y	Y	Y	Y	Y	Y	--	Y	Y	N	Y	Y	Y	Y	N	N	N
Imai et al, 2007(47)	Y	Y	Y	Y	N	N	Y	--	Y	Y	N	N	N	Y	Y	N	N	N
Lake et al, 2009(50)	Y	Y	Y	Y	N	Y	Y	--	Y	Y	N	Y	Y	Y	N	N	N	N
Radat et al, 2005(52)	Y	Y	Y	Y	Y	Y	Y	N	Y	Y	N	Y	N	N	N	N	N	N
Zidverc-Trajkovic etr al, 2007(54)	Y	Y	Y	Y	Y	Y	Y	--	Y	Y	N	Y	N	Y	Y	N	N	N
Chagas, 2015(43)	N	Y	Y	Y	Y	Y	Y	--	Y	Y	N	Y	N	N	N	N	N	N

Note: *abstract only

	STROBE Item Number															
Citation	13a	13b	13c	14a	14b	14c	15	16a	16b	16c	17	18	19	20	21	22
Articles reporting on treatment patterns among triptan users																
Chen, 2014(36)	N	N	N	Y	N	Y	Y	Y	--	--	Y	Y	Y	Y	Y	Y
Lombard, 2018(39)*																
Ng-Mak, 2012(40)	Y	Y	Y	N	N	N	Y	N	--	N	N	Y	Y	Y	Y	Y
Alam, 2018(35)*																
Fischer, 2016(37)	Y	N	Y	Y	N	N	N	Y	--	N	Y	Y	Y	Y	Y	Y
Serrano, 2013(41)	N	N	N	Y	N	N	N	Y	--	N	Y	Y	Y	Y	Y	Y
Katic, 2011(38)	Y	N	N	Y	N	N	N	N	--	N	Y	Y	Y	Y	Y	Y
Articles reporting on MOH among triptan users																
Katsarava et al, 2003(48)	Y	N	N	N	N	Y	Y	N	--	N	--	Y	Y	Y	Y	N
Kluo--itis et al, 2017(49)	Y	Y	Y	Y	N	N	N	Y	--	N	Y	Y	N	Y	Y	N
Limmroth et al, 2002(51)	Y	N	N	N	N	N	N	Y	N	N	Y	Y	N	Y	Y	N
Zeeberg et al, 2006(53)	N	N	N	N	N	N	N	Y	--	N	Y	Y	Y	Y	Y	Y
Benz et al, 2017(42)	Y	Y	Y	Y	N	Y	Y	Y	--	N	Y	Y	Y	Y	Y	N
Creac'h et al, 2009(44)	Y	N	N	Y	N	N	N	Y	--	N	Y	Y	Y	Y	Y	N
de Rijk et al, 2018(45)	Y	Y	Y	Y	N	N	N	Y	--	N	Y	Y	Y	Y	N	N
Grande et al, 2011(46)	Y	Y	N	Y	N	Y	Y	Y	--	N	Y	Y	N	Y	Y	Y
Imai et al, 2007(47)	N	N	N	Y	N	N	N	N	--	N	N	Y	N	Y	N	N
Lake et al, 2009(50)	N	N	N	Y	N	N	N	Y	--	N	Y	Y	Y	Y	N	N
Radat et al, 2005(52)	N	N	N	Y	N	N	N	Y	--	N	Y	Y	Y	Y	N	N
Zidverc-Trajkovic etr al, 2007(54)	N	N	N	Y	N	Y	N	Y	--	N	Y	Y	N	Y	Y	N
Chagas, 2015(43)	Y	N	N	Y	N	N	N	Y	N	N	Y	Y	N	Y	Y	N

Note: *abstract only
